# Comparison between pre-operative MRI and intra-operative endoscopic findings for idiopathic normal pressure hydrocephalus

**DOI:** 10.1186/2045-8118-12-S1-O40

**Published:** 2015-09-18

**Authors:** Daisuke Kita, Yasuhiko Hayashi, Issei Fukui, Masahiro Oishi, Cheho Park, Mitsutoshi Nakada

**Affiliations:** 1 Department of Neurosurgery, Noto General Hospital, Japan; 2 Department of Neurosurgery, Kanazawa University, Japan

## Introduction

Endoscopic examination of the intra-ventricular walls is rarely performed for cases of idiopathic normal pressure hydrocephalus (iNPH) since shunting is the first treatment option for iNPH. We conducted intra-operative endoscopy during shunt surgery in patients with iNPH and compared the findings with their pre-operative MRI data.

## Methods

Eleven patients (6 men and 5 women, age range: 67-84 years, mean age: 75.5 years) with probable iNPH consistent with the Japanese iNPH guideline were included in this study. High-resolution MRI (T1-3D-SPGR or FIESTA) was conducted pre-operatively. Intra-ventricular observations were performed with a flexible endoscope, Olympus VEF-type V, via a frontal burr hole during shunt surgery. Student's t-test and Fisher's direct method were employed for statistical analyses.

## Results

Laceration of the septum pellucidum was found in 4 patients (36.3%) by direct endoscopic observations. The foramen of Monro and Sylvian aqueduct were not stenosed in any case, as revealed both radiologically and endoscopically. For all cases, downward ballooning of the third ventricle floors was not observed in pre-operative MRIs, while thin third ventricular floor and lamina terminalis were observed in intra-operative endoscopic views. The interspace between the bilateral mammillary bodies varied from being wide to narrow, as revealed by endoscopy. A significant correlation was found between laceration of the septum pellucidum and the callosal angle measured by MRI (104 ± 5.77 degrees for lacerated vs. 70.3 ± 7.44 degrees for non-lacerated, p < 0.001). Width of the third ventricle and that of the interspace between the bilateral mammillary bodies showed a non-significant correlation (14.2 ± 1.86 for widely opened vs. 11.7 ± 2.49 for narrowly closed, p = 0.052).

## Conclusion

In iNPH, the pre-operative MRI findings of dull callosal angle and wide third ventricle were closely related to the intra-operative endoscopic findings of laceration of the septum pellucidum and wide opening between the bilateral mammillary bodies, respectively.
